# Developmental Differences in Prosocial Behavior Between Preschool and Late Elementary School

**DOI:** 10.3389/fpsyg.2019.00876

**Published:** 2019-04-26

**Authors:** Lisa Flook, Carolyn Zahn-Waxler, Richard J. Davidson

**Affiliations:** Center for Healthy Minds, University of Wisconsin-Madison, Madison, WI, United States

**Keywords:** prosocial, social decision-making, childhood, sharing, developmental change

## Abstract

Research points to evidence of innate prosocial tendencies present early in life. As more complex cognitive abilities emerge with development, this may alter the expression and nature of prosocial behaviors over time. Sharing is one important expression of prosocial behavior. Our aim was to explore how children’s sharing behavior with different recipients across important social categories changes by comparing two distinct transitional periods in development. We compared the responses of 46 preschoolers (*M* age = 4.95 years) and 52 5th graders (*M* age = 9.98 years) on two social decision-making paradigms. Results showed that older children shared more selectively depending on the recipient than younger children, who shared resources more equitably with different recipients. A second paradigm revealed greater uncoupling of behavior and cognition among older children, such that prosocial behavior in preschoolers was more closely linked to their judgments about the recipient’s character than it was for 5th graders. Increased cognitive complexity that emerges over the course of development can be used to help or discriminate against others, depending upon how those capacities are channeled. Therefore, how these abilities are shaped has important societal consequences.

## Introduction

The distribution of resources is a primary concern that shapes social exchanges and formation of society. Prosocial behaviors are a powerful force for promoting group cohesion and acceptance, and one individual who acts generously has the ability to influence others to behave more generously (Fehr and Fischbacher, 2003; Klapwijk and Van Lange, 2009; Fowler and Christakis, 2010; Layous et al., 2012). Conversely, one individual who behaves selfishly can lead others to defect and act more selfishly (Rand et al., 2009). Greater understanding of how children distribute resources across different social contexts and based on recipient characteristics offers insight into the mulitfacted development of prosocial behavior. This is important for understanding how to cultivate our vast human potential.

Research points to evidence of innate prosocial tendencies from an early age (Rheingold and Hay, 1980; Kärtner et al., 2010; Svetlova et al., 2010; Vaish et al., 2010; Hepach et al., 2012; Sloane et al., 2012). Prosocial behavior increases over the first 3 years of life (Knafo et al., 2008; Roth-Hanania et al., 2011). The core of concern for others is evident in young infants (Davidov et al., 2013). Infants show a preference for those who help rather than hinder another from attaining a goal (Hamlin et al., 2007). Likewise, toddlers display spontaneous helping behavior even in the absence of any personal reward or reciprocation, for example, displaying greater happiness when giving is costly to themselves (Aknin et al., 2012), and rewarding those who are more generous toward others. Children also take fairness and equity into account when making decisions about resource allocation (Blake and McAuliffe, 2011; Kanngiesser and Warneken, 2012) and make judgments about fairness from early on in life (Geraci and Surian, 2011).

Moreover, research highlights a natural benevolent tendency among adults whereby spontaneous generosity is displayed when making split-second decisions (Rand et al., 2012). However, when primed to engage rational thought or given more time to ponder decisions, people are less generous. This finding suggests that certain forms of cognition can play a role in hindering sharing behavior. As such, one hypothesis might be that with cognitive maturation (and the attendant increase in capacity for complex, higher level mental operations), children become less generous as they age.

On the other hand, the ability to act in less self-focused ways, and thus display greater generosity, may increase with cognitive development as the capacity for more advanced forms of perspective taking, including the ability to inhibit selfish impulses, and to exert greater self-control come online (Rueda et al., 2004; Diamond, 2006). Indeed, older adolescents and young adults display more reciprocity and show greater activity in brain regions associated with perspective taking as compared to younger adolescents (Van den Bos et al., 2011). As such, the role of developmental maturation and capacity for complex, higher order cognition on prosocial behavior appear somewhat contradictory. On the one hand, engaging rational cognitive processes can reduce generosity, whereas, on the other, development enables advanced forms of cognition that can enhance prosociality. Does this mean that over the course of development, we become more or less generous? Do younger children with less developed cognitive complexity behave more altruistically? Or does the development of greater cognitive control enable older children to inhibit selfish impulses and act more generously?

Research by Fehr and colleagues suggests that sharing does not emerge until later in childhood and that a strong preference for egalitarian outcomes is also displayed (Fehr et al., 2008). However, others suggest that sharing behavior emerges earlier in childhood dependent upon the recipient (Moore, 2009). The latter study, though, did not include older children; therefore, it was not possible to examine developmental changes in sharing with different recipients. While both studies utilized forced-choice paradigms, decisions in the real world are often more fluid. Forced-choice tasks have served as a useful paradigm for assessing and comparing human behavior with nonhuman primates (Warneken et al., 2007; House et al., 2012). But they may also underestimate both nonhuman and human’s actual ability and willingness to share, as suggested by evidence of young children’s sharing behavior given an open-choice response format (Benenson et al., 2007) and as seen in naturalistic contexts (Conte et al., 2018). Others have also noted the limitations of a forced-choice paradigm with predefined allocation options (Warneken et al., 2011).

In another study of costly sharing, in which recipients give up resources for themselves when they give to a recipient, researchers compared sharing behavior of 4- and 8-year-old children in response to characteristics of hypothetical recipients (Malti et al., 2016). Children were free to choose how much to share, as there were no predefined choices in this study. Children shared more with recipients who were described as morally deserving and needy. However, 8-year-olds shared less with morally undeserving recipients, indicating greater differentiation in sharing with age based on recipient characteristics.

In the current study, we were interested in two aspects of prosocial behavior. The first pertained to the social contexts in which children share with others. Our social groups tend to be defined by an in-group comprised of those with whom we affiliate, invest and share resources, while others fall into an out-group. There is a tendency to share more with members of our in-group than out-group because of social bonds and norms of reciprocity, which involve giving and receiving physical and psychological resources within our in-group, whereas, we are more likely to withhold and compete for resources with those perceived to be in the out-group (Tajfel, 1982). The out-group can be defined in different ways, including those who are unfamiliar (neutral strangers), those who are familiar but disliked, and those who are unfamiliar but vulnerable (and merit our support). Of particular interest is prosocial behavior toward out-group members, which holds significance for intergroup relations – more widespread expressions of such humanity could promote greater peace and harmony, significantly altering our society.

To examine prosocial behavior within a range of social contexts, we looked at sharing across different recipients. A sharing task with an open-choice format was used in order to detect possible gradations in prosocial responses. The four types of recipients included in-group (i.e., most liked peer) and out-group (i.e., least liked peer, stranger, sick child) targets. We examined differences in sharing on separate trials in the four recipient groups by comparing the amount of resources retained versus given to each target. We expected younger children to share more equitably across recipients and older children, who have greater capacity for complex reasoning, to show more discrimination and differentiation, as reflected in less sharing with out-group members who are disliked, but more sharing with those in need.

Our second interest was in examining how children’s prosocial behavior is related to their social attributions and biases in judgment. Prior research shows that even minimal information about trivial circumstances influences social perceptions, namely attributions about the character of those who experience lucky versus unlucky events (Olson et al., 2006, 2008). Across cultures, adults and children as young as 3 years old judge someone who experiences a minor lucky event more favorably than someone who experiences a comparable unlucky event – those judgments also extend to their associates – revealing a systematic bias in social judgment based on minimal information and happenstance that generalizes to others who belong to the same group. In this way, what appears to be an innocuous preference for the lucky could play a role in the development and maintenance of biases against disadvantaged people and groups (Olson et al., 2008).

We extend this paradigm to explore the intersection between cognition (social judgments) and prosocial behavior (giving choices) by examining whether this bias in judgment against those who experience minor unlucky events also influences subsequent behavior toward those individuals. First, we presented a small gift for children to give to one of the targets, and then queried children about their attributions of the target, in order to examine how children’s social judgments are linked to their prosocial behavior (giving a gift). While social judgments may influence giving, with increased cognitive capacity, greater uncoupling of cognition and behavior becomes possible, such that younger children are expected to give to those they judge more favorably, whereas, older children are expected to show greater differentiation between their social judgments and giving.

## Methods

### Sample

Children were recruited from 4-year-old kindergarten (Pre-K) and 5th grade general education classrooms as part of a larger project investigating social skills development in an urban public school district in the Midwest. These two grade levels enabled comparison of children before they have much exposure to formal schooling (both academic instruction and social norms) and later in childhood when children are nearing completion of elementary school education and the capacity for more complex and abstract mental representations begins to emerge.

Letters describing the project were sent home to parents with a consent form inviting participation. Research was approved by the Education and Social/Behavioral Sciences IRB at UW-Madison. Children whose parents provided written informed consent and who themselves gave assent completed the sharing and attributional tasks in individual testing sessions. The Pre-K sample consisted of 46 children (31 boys, age: *M* = 4.95, SD = 0.34, range: 4.44–5.51) and the 5th grade sample consisted of 52 children (31 boys, age: *M* = 9.98, SD = 0.35, range: 9.38–10.91). The ethnic composition of the sample was as follows for preschoolers: 70% Caucasian, 11% Hispanic, 9% African American, 4% Asian, and 6% other; and for 5th grade students: 27% Caucasian, 44% Hispanic, 13.5% African American, 2% Asian, and 13.5% other. Among preschoolers, level of parent education consisted of 65% with a college degree or higher, 35% had some college degree or less; for 5th grade students, parent level of education for 40% was a college degree or higher, 58% had some college degree or less (1 participant did not report this information). Note, ethnicity and parent education were controlled for in the analyses presented in this paper. For analytic purposes, ethnicity was coded Caucasian and non-Caucasian given the composition of the sample. The social contexts for sharing (aka “sharing”) and attributions and giving (aka “lucky”) tasks were administered in two separate testing sessions, with the lucky task completed on the first day and the sharing task on the second day.

### Measures

#### Social Contexts for Sharing Task

The sharing task consisted of four separate trials in which children distributed stickers between themselves and a target recipient for each trial. This task is a variation of the dictator game, a behavioral economics paradigm designed to measure altruism and fairness in adults. Other versions of the dictator game have been successfully adapted for use with children (e.g., Gummerum et al., 2010). Before the task began, children were presented with six different types of stickers and asked to select the four types they liked best. On each of the four trials, a different sticker type from among those selected by the child was used for variety and novelty in order to maintain interest and reduce habituation. The four target recipients included the peer from their class who the participant identified as liking the most and least, an unfamiliar child, and a child who was sick. Pictures representing each of these four recipients were adhered to an envelope. An actual photo was used of the most and least liked child, a picture of a silhouette of a same-gendered child was used to represent the stranger, and a picture of a same-gendered child with a thermometer (girls) or ice pack (boys) represented the sick child. In each of the four trials, children were presented with an envelope for themselves labeled “me” and an envelope for the designated target recipient with the corresponding picture on the envelope. Children were given 10 stickers at the beginning of each trial and told they could keep as many as they would like for themselves and give as many as they would like to the other person. The examiner turned away while the child distributed the stickers. At the end of the task, the examiner placed all of the stickers from the “me” envelopes into a single envelope for the child to take with them and after the child left, the stickers from each of the other envelopes was counted. The number of stickers given to each of the four recipients is the sharing score, ranging from 0 to 10 for each recipient.

#### Attributions and Giving Task (or Lucky Task)

Scenarios depicting minor lucky and unlucky occurrences were adapted from Olson et al. (2008). The task was originally developed to provide insight into how judgments about social groups are formed by exploring developmental and cultural manifestations of the tendency to evaluate others based on unintentional circumstances. On each of nine trials, a description was presented of a child who experienced a lucky event paired with another child who experienced an unlucky event. The verbal descriptions were accompanied by a drawing of the scenarios with the target child’s gender in each scenario matched to the participant. First, children were presented with a small wrapped gift box and asked to decide which target should receive the gift. Next, children were asked which of the target children is nicer. Lucky and unlucky items were counterbalanced in order of presentation. Responses were coded 1 for lucky and 2 for unlucky. Examples of lucky events included finding an extra hour of cartoons on TV, playing soccer on a sunny day, and seeing a rainbow in the sky. Examples of unlucky events included stepping in a puddle of mud, money getting stuck in a candy bar machine, and getting hit by a pinecone that fell from a tree.

### Analysis Plan

Developmental differences in prosocial behavior were examined in a sample of 98 children from preschool (*n* = 46) and 5th grade (*n* = 52). Grade level differences in sharing and lucky tasks were examined with multivariate General Linear Model (GLM) in two separate models, each controlling for ethnicity, parent education, and gender. The multivariate approach was used to reduce the number of analyses by entering multiple outcomes from each task simultaneously in the same model. Four outcomes were simultaneously entered for the sharing task model and two outcomes were simultaneously entered for the lucky task model. RMANOVA was used to examine interactions between grade level and recipients on the sharing and lucky task in a separate model for each task. Partial eta squared ($\eta_{p}^{2}$) was computed to estimate the variance in the outcome associated with the grouping variable of interest while partialling out the effect of other control variables (Richardson, 2011). Item level differences between behavior (giving gift) and niceness attributions on lucky task trials were analyzed by grade level using a *t*-test. Gender differences are reported where significant. Results are presented first for the sharing task followed by analyses of the lucky task.

## Results

### Social Contexts for Sharing Task

**Figure 1:**
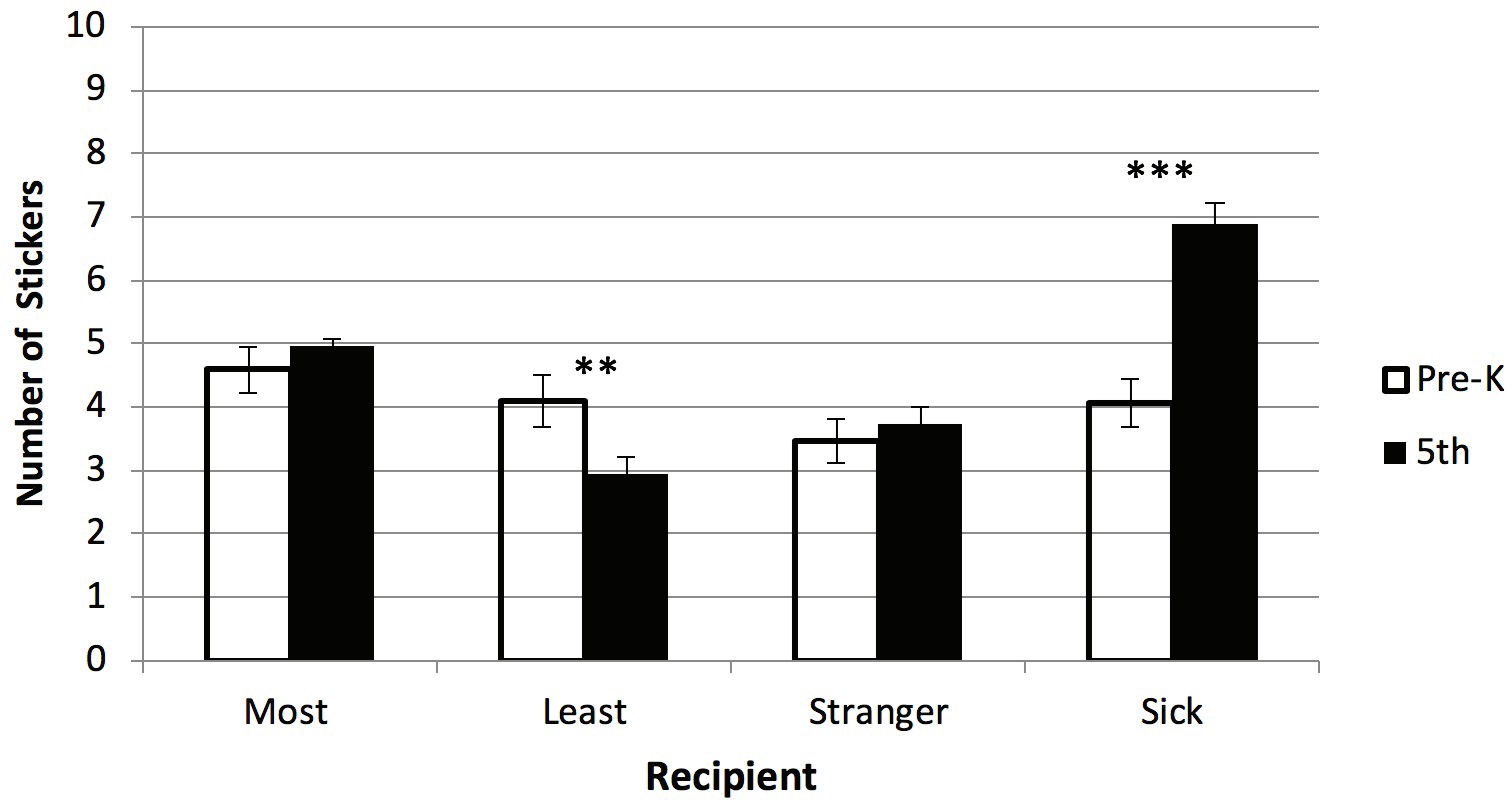
Developmental differences in resource allocation across recipients on Sharing Task. Younger children (Pre-K) shared more stickers with the peer they like least as compared to older children (5th grade). Older children shared more stickers with a child who is sick as compared to younger children. **p < 0.01, ***p < 0.001.

Differences by grade level on the sharing task were examined in a multivariate GLM across four outcomes (representing the average number of stickers given to the most liked peer, least liked peer, stranger, and sick child). Descriptive statistics for the sharing task are presented in Table 1. There were significant differences in sharing by grade level controlling for ethnicity, parent education, and gender (*F*(4,81) = 10.30, *p* < 0.001). More specifically, significant differences emerged for sharing with the least liked peer and sick child (see Figure 1). Younger children gave more stickers on average to the peer they identified as least liked (*F*(1,84) = 7.35, *p* = 0.008, $\eta_{p}^{2}$ = 0.08) compared to their older counterparts. Older children shared more stickers with the sick child (*F*(1,84) = 27.25, *p* < 0.001, $\eta_{p}^{2}$ = 0.25) as compared to younger children. There were no significant differences in sharing by ethnicity or parent education. Girls shared more than boys when the target recipient was the person they liked least (*F*(1,84) = 6.22, *p* = 0.015, $\eta_{p}^{2}$ = 0.07; girls: *M* = 4.14, SD = 1.93, boys: *M* = 2.98, SD = 2.48). No significant gender by grade level interactions emerged.

**Table 1:** Descriptive statistics for sharing task and lucky task by grade level.

	Pre-K		5th Grade	
	Mean	SD	Mean	SD
**Sharing task**				
Most liked	4.59	2.32	4.96	0.84
Least liked	4.10	2.61	2.94	1.96
Stranger	3.46	2.26	3.75	1.71
Sick child	4.07	2.41	6.90	2.26
**Lucky task**				
Gift giving	1.42	0.31	1.91	0.19
Nicer child	1.41	0.28	1.60	0.27

Notes: The possible range for the Sharing Task average on each trial is 0–10, with higher values indicating that more stickers were given to the recipient. For the Lucky Task average, the possible range is 1–2, with values closer to 1 indicating that the lucky child was selected, whereas, values closer to 2 indicate that the unlucky child was selected.

**Figure 2:**
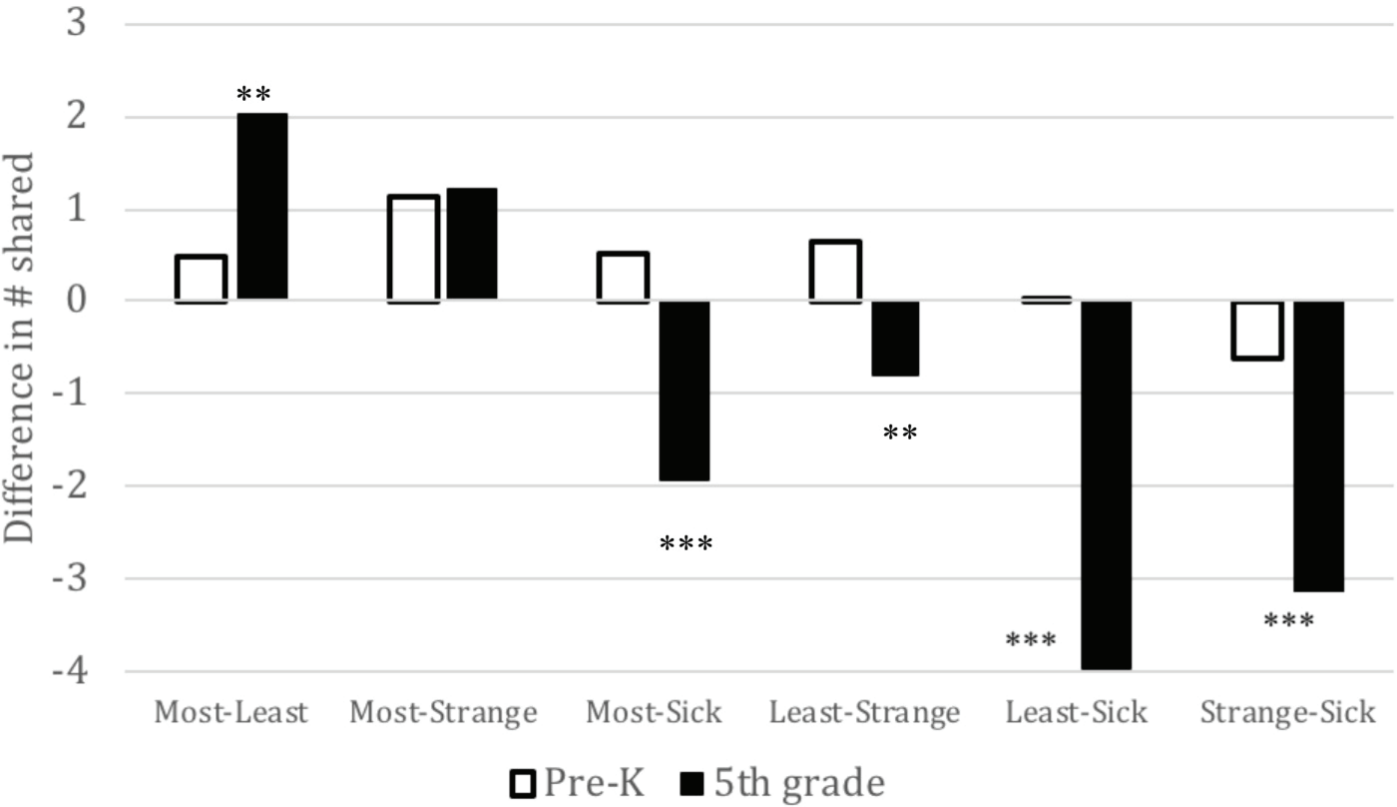
Developmental differences in amount of sharing by pairs of recipients. Amounts were calculated by subtracting (recipient 1 – recipient 2) as shown in order listed. There was a significant interaction between grade level and recipient with grade level differences in amount of sharing between the following pairs of recipients: (1) Most-Least, older children gave about 1.5 stickers more than younger children did to their most liked peer compared to their least liked peer; (2) Most-Sick, older children gave about 2.5 stickers more than did younger children to a sick child compared to their most liked peer, (3) Least-Strange, older children gave about 1.25 stickers more to a stranger compared to their least liked peer than did younger children, (4) Least-Sick, older children gave about 4 more stickers to a sick child than their least liked peer, as compared to younger children, (5) Strange-Sick, older children gave about 2.5 more stickers to a sick child than a stranger as compared to younger children. **p < 0.01, ***p < 0.001.

Repeated measures ANOVA (RMANOVA) was used to examine grade level by recipient interactions for the sharing task. There was a significant interaction of grade level and sharing between recipient pairs (Wilks’ Lambda: *F*(3,82) = 13.21, *p* < 0.001) controlling for ethnicity, parent education, and gender. Tests of within-subjects contrasts were significant for five out of six pairs of recipients, indicating that younger and older children shared differentially. Overall, older children exhibited greater differentiation in sharing among different recipients as compared to younger children (see Figure 2).

#### Most-Least

Older children showed a bigger difference than younger children in giving to their most liked peer than their least liked peer (*F*(1,84) = 8.16, *p* = 0.005, $\eta_{p}^{2}$ = 0.09). Older children gave approximately two more stickers to their most liked peer than their least liked peer, whereas younger children gave approximately a half a sticker more to their most liked peer than their least liked peer.

#### Most-Stranger

There was no grade level difference in sharing between the most liked peer and a stranger.

#### Most-Sick

Older children showed a different pattern of giving for a sick child and their most liked peer compared to younger children (*F*(1,84) = 14.19, *p* < 0.001, $\eta_{p}^{2}$ = 0.15). Older children gave approximately two stickers more to a sick child, whereas, younger children showed a preference for most liked peer, giving approximately one-half sticker more to their most liked peer than a sick child.

#### Least-Stranger

In the case of sharing with a stranger and least liked peer, older children gave differently than younger children (*F*(1,84) = 5.60, *p* = 0.002, $\eta_{p}^{2}$ = 0.06). Older children gave more to a stranger than least liked peer, by about three-quarters of a sticker, whereas younger children gave more to their least liked peer than a stranger, by about one-half sticker.

#### Least-Sick

Older children differentiated between giving to a sick child and their least liked peer compared to younger children (*F*(1,84) = 36.58, *p* < 0.001, $\eta_{p}^{2}$ = 0.30). Older children gave about four stickers more to a sick child, while younger children did not differentiate, giving about the same amount to a sick child and their least liked peer.

#### Stranger-Sick

Children gave more to a sick child than a stranger in both grades, but the difference was greater among older children (*F*(1,84) = 23.98, *p* < 0.001, $\eta_{p}^{2}$ = 0.22). Older children gave over three stickers more to a sick child than a stranger, whereas younger children gave only about half a sticker more to a sick child.

### Attributions and Giving Task (or Lucky Task)

**Figure 3:**
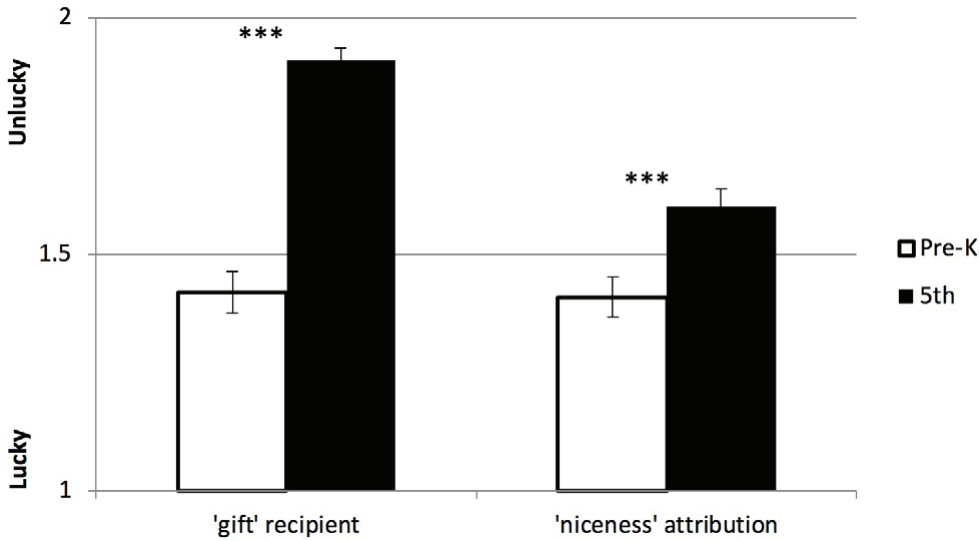
Developmental differences in attributions and giving on Lucky Task. Older children (5th) gave a present to the unlucky child more often and judged the unlucky child as nicer compared to younger children (Pre-K). ***p < 0.001.

Differences by grade level for the lucky task were examined in a multivariate GLM with two outcomes specified (average number of trials the unlucky child was selected to receive gift, and average number of trials the unlucky child was judged to be nicer). There were grade differences in both behavior and attributions controlling for ethnicity, parent education, and gender (*F*(2,89) = 40.5, *p* < 0.001). Older children gave the present to the unlucky child more often (*F*(1,90) = 81.70, *p* < 0.001, $\eta_{p}^{2}$ = 0.48) and judged the unlucky child to be nicer (*F*(1,90) = 11.98, *p* = 0.001, $\eta_{p}^{2}$ = 0.12) as compared to younger children (see Figure 3). There were no differences by ethnicity, parent education, or gender, and no gender by grade level interactions were found for giving behavior and niceness ratings on the lucky task.

Using RMANOVA, a significant interaction between grade level and trial type emerged (*F*(1,90) = 23.22, *p* < 0.001, $\eta_{p}^{2}$ = 0.21), indicating that there were differences by grade level in the association between giving behavior and niceness attributions, controlling for ethnicity, parent education, and gender. The association was further examined by comparing the correlation between the giving and niceness variable within each grade. The correlation for younger children (*r* = 0.57, *p* < 0.001) was significant, whereas it was not significant for older children (*r* = 0.20, *p* = 0.16), indicating that for younger children, giving behavior and attributions of niceness were more closely aligned. In addition, the magnitude of the correlations for the younger and older children significantly differed (*z* = 2.13, *p* = 0.033).

**Figure 4:**
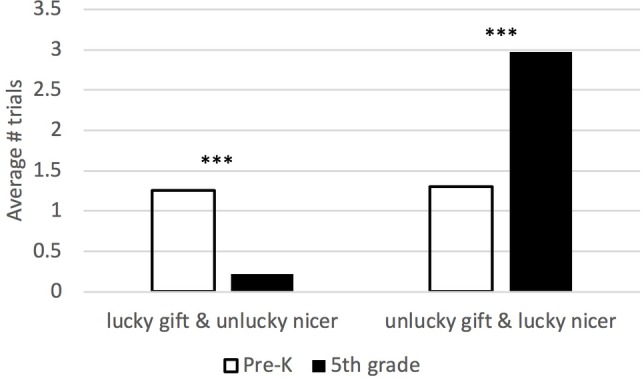
Developmental differences in divergence between attributions and giving. There was a significant interaction between grade level and trial type such that younger children more often gave to the lucky child but judged the unlucky child as nicer (on average, approximately one out of nine trials), compared to older children (who rarely exhibited this combination of “lucky gift & unlucky nicer”). Older children more often gave to the unlucky child but judged the lucky child as nicer (on average, approximately three out of nine trials), compared to younger children (who exhibited this combination of “unlucky gift & and lucky nicer,” on average, approximately one out of nine trials). ***p < 0.001.

In order to further examine how patterns of giving and attributions diverged on the lucky task, we assigned a score by subtracting the niceness response from the giving choice for each of the nine trials. This resulted in three possible scores: a difference score of +1 indicates that the child gave to the unlucky target but named the lucky target as nicer on that trial, a score of 0 indicates that the child gave the gift and attributed niceness to the same target on that trial, and a score of −1 indicates that the child gave to the lucky target but named the unlucky target as nicer on that trial. These scores were tallied and an average count score for each category was computed and compared by grade level with an independent *t*-test. As shown in Figure 4, younger children more often chose to give the gift to the lucky child, but identified the unlucky child as nicer (younger: *M* = 1.26, SD = 1.89; older: *M* = 0.22, SD = 0.58; *t*(96) = 3.81, *p* < 0.001). On average, younger children gave to the lucky child but judged the unlucky child as nicer, on approximately one out of nine trials, compared to older children who only exhibited this combination choice on one-fifth of one trial out of nine trials. Whereas, older children more often gave the gift to the unlucky child, but named the lucky child as nicer (younger: *M* = 1.30, SD = 2.00; older: *M* = 2.97, SD = 2.45; *t*(96) = −3.64, *p* < 0.001). On average, older children gave to the unlucky child but judged the lucky child as nicer on approximately three out of nine trials, compared to younger children, who exhibited this combination choice, on average, in approximately one out of nine trials. There was not a significant difference between groups in giving and attributing niceness to the same child.

## Discussion

Decisions regarding sharing and allocation of resources, amid the complex array of social relationships that exist in the world, have important social and economic consequences. Prior evidence suggests diverging predictions with regard to the role of development on these aspects of prosocial behavior. Therefore, we observed how children make decisions about distributing resources in different circumstances and how development impacts those decisions. We examined giving behavior within two distinct age groups, preschool and older elementary school children, in order to observe developmental differences. In one set of scenarios, giving involved incurring a cost to oneself in relation to sharing with a variety of other recipients, and in another condition, giving did not involve any cost to oneself. The second paradigm (social decision-making) also allowed us to examine congruence between social attributions and prosocial behavior.

Older children as compared to younger children appear to use more complex reasoning in determining resource allocation. Rather than displaying a robust preference for egalitarian outcomes, older children make choices that depend on the social category of the recipient. This outcome is consistent with research by Malti et al. (2016) who found that 8-year-olds showed more differentiation in sharing resources with hypothetical recipients who varied on how needy and morally deserving they were, as compared to 4-year-olds. These differences highlight the context-dependent nature of sharing and the importance of taking into account various dimensions of relationships that comprise our social sphere.

Sharing with close others emerges relatively early and remains stable, as both older and younger children share similarly with the peer they like most. However, sharing with those who are in need (or in challenging circumstances) appears to develop later, as does the tendency to withhold resources from a least liked peer. Compared to younger children, older children give less to those they least like. However, older children give more to a child who is sick than do younger children, which may reflect greater perspective taking. Notably, both younger and older children share at least a small amount with everyone, including those who are unfamiliar and those who they least like. This is in line with other research that shows children are willing to incur some cost to themselves even when there is little apparent incentive to behave prosocially (Fehr et al., 2008). Helping children at both (and all) ages to enhance this aspect of their being is an important future goal. In terms of gender differences, girls tend to show less of an out-group bias, as they behave more generously toward a least liked peer than do boys. The tendency for girls to give more has been found in other research, and may stem from socialization practices and societal norms that place an emphasis on generosity and caretaking for girls (Gummerum et al., 2010).

In general, older children showed more differentiation in resource distribution across recipients as compared to younger children, as predicted by our hypothesis. The main distinctions younger children make in resource allocation are between the peer they like most and someone who is unfamiliar. Older children on the other hand display a more nuanced capacity to take into account the particular recipient, giving more or less accordingly. For example, older children shared more with a sick child than anyone else including the peer they like most. On average, older children gave five times more than younger children did to a sick child as compared to their most liked peer. But older children shared less with the peer they liked least as compared to everyone else, preferring to share more even with a stranger. Older children gave four times more than younger children to their most liked peer compared to their least liked peer, and seven times more to a stranger than their least liked peer.

Confirming our second hypothesis, older children were more generous toward unlucky others and more likely to judge them as nice than younger children. Whereas, there was stronger convergence between sharing and attributions among younger children. That is, younger children do what they think (or think what they do), as evidenced by a significant correlation between attributions and giving. On the other hand, for older children thinking and doing don’t necessarily correspond, as indicated by a nonsignificant correlation between attributions and giving, in addition to the tendency for older children to display giving toward an unlucky child, while judging the lucky child as nicer. Thus, with development, older children evidence an ability to hold contradictory beliefs and actions. This uncoupling process may also reflect the ability to compartmentalize as children grow older.

This study built on prior research by comparing two developmentally distinct age groups. While there were demographic differences in the sample composition among the age groups, we controlled for these variables in analyses. Another limitation of the current study design is the inability to determine at which age differences emerge. Future research in a longitudinal sample or a larger cross-sectional sample with more age groups would help to clarify the developmental trajectory. An interesting extension of this work would be to examine how patterns of giving behavior are associated with children’s values and adjustment over time. Research suggests that a focus on materialistic values is associated with lower levels of well-being (Kasser, 2002). In addition, including measurement of skills related to emotion (understanding and response), along with cognition and executive functions would help to elucidate the mechanisms underlying developmental differences in prosocial behavior.

The apparent paradox of development is that older children have a better ability to make distinctions, among people as well as between their behavior and cognition, leading at times to more helpful and other times more hurtful behavior. For older children, decisions reflect greater consideration of the individual recipient and a higher degree of uncoupling between cognition and behavior. Older children are willing to tolerate inequitable distributions that favor another who is disadvantaged (sick or unlucky). However, they are also more discriminating against the peer they like least. This may be related to the development of exclusionary behaviors like prejudice and contribute to social inequities through an imbalanced distribution of resources.

In conclusion, with higher cognitive capacities and more complex reasoning developing over the course of childhood, discernment is possible, but also discrimination. Therefore, how these abilities are shaped or trained has important consequences for societal evolution. Evidence points to the capacity to increase prosocial tendencies in children with training at an early age (Izard, 2002; Schonert-Reichl et al., 2004; Flook et al., 2015). Deliberately cultivating generosity and related qualities such as empathy, kindness, forgiveness, and compassion through education may have the potential to transform and channel developing capacities in ways that benefit humanity as a whole.

## Ethics Statement

UW-Madison Education and Social/Behavioral Sciences IRB Written consent was provided by children’s parents and children provided assent for their participation.

## Author Contributions

LF and RD designed the study. LF analyzed the data and all authors discussed the interpretation of data. LF and CZ-W contributed to writing the paper with comments from RD.

### Conflict of Interest Statement

The authors declare that the research was conducted in the absence of any commercial or financial relationships that could be construed as a potential conflict of interest.
